# The Natural History of Biopsy-Negative Rejection after Heart Transplantation

**DOI:** 10.1155/2013/236720

**Published:** 2013-12-18

**Authors:** Zhaoyi Tang, Jon Kobashigawa, Matthew Rafiei, Lily Kagan Stern, Michele Hamilton

**Affiliations:** Cedars-Sinai Heart Institute, 127 South San Vicente Boulevard, Third Floor Cardiology, A3107, Los Angeles, CA 90048, USA

## Abstract

*Purpose.* The most recent International Society for Heart and Lung Transplantation (ISHLT) biopsy scale classifies cellular and antibody-mediated rejections. However, there are cases with acute decline in left ventricular ejection fraction (LVEF ≤ 45%) but no evidence of rejection on biopsy. Characteristics and treatment response of this biopsy negative rejection (BNR) have yet to be elucidated. *Methods.* Between 2002 and 2012, we found 12 cases of BNR in 11 heart transplant patients as previously defined. One of the 11 patients was treated a second time for BNR. Characteristics and response to treatment were noted. *Results.* 12 cases (of 11 patients) were reviewed and 11 occurred during the first year after transplant. 8 cases without heart failure symptoms were treated with an oral corticosteroids bolus and taper or intravenous immunoglobulin. Four cases with heart failure symptoms were treated with thymoglobulin, intravenous immunoglobulin, and intravenous methylprednisolone followed by an oral corticosteroids bolus and taper. Overall, 7 cases resulted in return to normal left ventricular function within a mean of 14 ± 10 days from the initial biopsy. *Conclusion.* BNR includes cardiac dysfunction and can be a severe form of rejection. Characteristics of these cases of rejection are described with most cases responding to appropriate therapy.

## 1. Introduction

Heart transplantation continues to provide patients with end-stage heart disease with extended survival with a half-life of 9.3 years between 2000 and June 2008. [1]. However, despite substantial advancements in immunosuppression, patients continue to be at significant risk for allograft rejection early after cardiac transplantation. The two recognized forms of allograft rejection are acute cellular rejection and antibody-mediated rejection (AMR). While acute cellular rejection has historically been the most common cause of allograft dysfunction, AMR has only recently become widely accepted [2].

During the 2004 International Society of Heart and Lung Transplantation (ISHLT), cellular rejection grades were revised and AMR was formally defined [3]. In April 2010, a publication from the ISHLT Consensus Conference assessed the status of AMR in heart transplantation and a pathologic grading scale was devised [4].

Despite the advent of new technology, such as gene expression profiling and echocardiograms, endomyocardial biopsy remains the standard for detecting rejection. To minimize the risk of a false negative, multiple specimens (usually 3–5) are obtained from 3 different sites. Though rare, false negatives do exist either through sampling error, artifact, Quilty lesions, or pathology misread.

Prior to the 2010 Consensus Conference, hemodynamic compromise, in the absence of acute cellular rejection, was termed biopsy-negative rejection. 10 to 20% of cardiac allograft recipients were diagnosed as having such rejection. Although the prevalence of the so-called biopsy-negative rejection has declined with the clinical diagnosis of AMR, there are incidences of patients who present with cardiac dysfunction (left ventricular ejection fraction, LVEF ≤ 45%) but have no biopsy findings of cellular or antibody-mediated rejection. These rejection episodes are now termed biopsy-negative rejection (BNR).

Since the outcome of patients with BNR (which includes cardiac dysfunction) has not been well established, we sought to review the characteristics and treatment response of patients who have developed BNR after heart transplantation.

## 2. Methods and Statistics

We retrospectively reviewed our cohort of patients who underwent heart transplantation between 2002 and 2012 and found 12 cases of BNR in 11 heart transplant patients, as defined by patients presenting with cardiac dysfunction, characterized by LVEF ≤ 45%, and who had no biopsy findings of cellular rejection or AMR. As severe infections are also known to cause left ventricular dysfunction, patients with a clear clinical picture of sepsis with fever, increase in white blood count, or positive cultures were excluded. Baseline characteristics and immunosuppression were collected and summarized. Characteristics and response to treatment at 90 days were noted. Continuous variable was presented as mean ± standard deviation while categorical variable is presented as percentages.

## 3. Results

We identified 11 patients who underwent heart transplantation between 2002 and 2012 and were treated for BNR. One of the 11 patients was treated a second time for BNR. The demographics of patients presenting with BNR was shown in Table 1. None of the BNR patients had previous blood transfusion or previous transplant. 5 (45%) patients had a previous VAD placement and 1 (9%) patient was African American. The baseline immunosuppression medications are as follows: 5 patients (45%) underwent induction therapy with antithymocyte globulin (ATG), 9 patients (82%) initiated with tacrolimus and mycophenolate mofetil, 1 patient (9%) initiated with cyclosporine and mycophenolate mofetil, and 1 patient (9%) initiated with cyclosporine and azathioprine.

The immunosuppression regimen at time of BNR onset is shown in Table 2. 2 patients switched to cyclosporine from tacrolimus because of reduced seizure threshold; 1 patient switched to a renal sparing protocol of sirolimus with mycophenolate mofetil because of renal function concerns. The 12 cases of BNR presented with an average LVEF of 34% ± 10% and 11 (92%) treated cases occurred during the first-year after transplant (Table 3). The mean time to the first incidence of BNR is 7.8 ± 7.5 months and no patient had treated rejection before onset of BNR except for the patient at the second onset. Among the 106 biopsies that have been done before BNR, 24 (23%) demonstrated low grade acute cellular rejection (1R, 1A, or 1B) and 1 (0.9%) showed suspicious antibody-mediated rejection (AMR 1). De novo circulating antibodies developed in 3 cases (in 3 patients) and 1 of them had donor-specific antibodies. 4 out of the 12 cases presented with LVEF ≤ 35% and presented with heart failure symptoms. Of these 4 heart failure cases, 2 required inotropic support—both received intravenous (IV) methylprednisolone (500 mg–1000 mg per day for 3 days), ATG (125–150 mg per day for 3–5 days), and IV immunoglobulin (IVIG, 70 g per day for 3 days), followed by oral corticosteroids (prednisone 80–100 mg per day bolus and taper). For the remaining 2 patients who presented with heart failure symptoms but did not necessitate inotropes, 1 was treated with ATG and IV methylprednisolone followed by prednisone bolus and taper and the other was given IV methylprednisolone followed by prednisone only. For the 8 out of 12 cases without heart failure symptoms, 7 were treated with only high dose oral corticosteroids. The remaining case was empirically treated with IVIG for two days.

**Figure 1:**
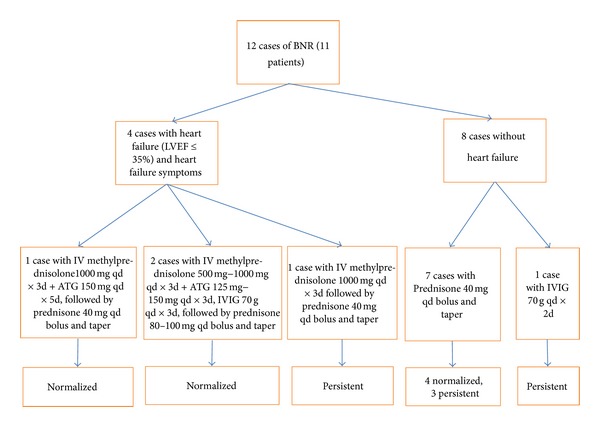
BNR treatment and effect flow chart.

Overall, 7 cases (58%) (in 7 patients) favorably resulted with return to normal left ventricular function (LVEF of 57% ± 6%) within a mean of 14 ± 10 days from the initial negative biopsy. Of these 7 patients, one expired approximately 6 months after the date of the normalized LVEF and another one expired approximately 4 months after the date of normalized LVEF. The remaining 5 cases (in 5 patients, including 1 patient's second case of BNR) maintained persistent left ventricular dysfunction beyond 90 days. A flow chart of treatment and effect could be found in Figure 1.

In summary, the LVEF status at 90 days is as follows: 5 cases (in 5 patients, including 1 case as second BNR on a patient) had persistent LV dysfunction, and 7 cases (in 7 patients) experienced normalized LVEF.

## 4. Discussion

As our understanding of the biological mechanisms underlying cardiac allograft rejection increases, the number of undiagnosed rejection decreases. The concept of BNR has evolved from its initial definition as hemodynamic compromise in the absence of acute cellular rejection to its current definition of patients presenting with cardiac dysfunction in the absence of biopsy findings of cellular rejection or AMR [2]. AMR has changed from being suggested as BNR to its own ISHLT biopsy grading scale. However, even though the majority of rejection episodes of unclear etiology have now been resolved to be cases of AMR, there are still cases where the biopsy findings are inconsistent with the clinical prognosis, that is, patients with a decrease in LVEF with no biopsy findings of rejection, cellular, or humoral.

Due to the inconsistencies of endomyocardial biopsies, the existence of BNR is questioned. This argument is furthered by the fact that prior to the 2010 AMR Consensus Conference, 10–20% of cardiac allograft recipients were diagnosed with BNR when in actuality the majority of patients most likely experienced AMR. This brings up the question of whether BNR should exist as a separate category of rejection or is merely a by-product of the problems inherent with endomyocardial biopsies. In one case study, sampling error or nonuniformity of histopathologic changes resulted in a false-negative biopsy [5]. In this case study, a heart transplant recipient with repeatedly unremarkable endomyocardial biopsies and a negative evaluation for humoral rejection was found to have severe subepicardial myocyte necrosis with classic cellular rejection in the subsequent autopsy. The subendomyocardial layer was free from rejection. This is contrary to other studies which have found that rejection is evenly distributed throughout the right ventricular endomyocardium [6].

Although endomyocardial biopsy is the primary resource to diagnose acute rejections in all the cases discussed in this study, there are a few noninvasive diagnostics that have been demonstrated to be helpful in diagnosing acute allograft rejection after heart transplantation. In one multicenter clinical trial, Pham et al. reported that using gene-expression profiling to monitor rejection for patients 6 months after heart transplantation was not associated with increased risk of serious adverse outcomes and could reduce the need for biopsy [7]. In two pilot studies, Wu et al. applied cardiac magnetic resonance (CMR) in vivo to detect immune-cell infiltration at sites of rejection by monitoring macrophages. The investigators subsequently developed a functional index from local strain analysis and proved it to be correlated with rejection grades [8, 9]. Although clinical applications have not been implemented, CMR was demonstrated to be capable of providing the rejection status of whole-heart perspective, and thus might be a potential tool of optimizing diagnosis of BNR. Another potential tool is speckle-tracking 2-dimensional strain echocardiography (2DSE). By using rat cardiac transplantation models, Pieper et al. demonstrated that 2DSE was able to differentiate myocardial function between rejection in allografts and nonrejection in isografts. Therefore 2DSE might potentially help with clinical practice in terms of early rejection monitoring [10].

Perhaps BNR is another form of AMR but due to the “newness” of AMR and the lack of a complete understanding of AMR, no definite conclusions can be made [11]. Despite the ISHLT revised biopsy rejection scale, inconsistencies of histologic light-microscopic features make recognizing AMR difficult [12].

This revised definition of BNR has been noted to result in a decrease in 3-year subsequent survival, lower subsequent freedom from cardiac allograft vasculopathy (CAV), and a decrease in freedom from nonfatal major adverse cardiac events (NF-MACE) [13]. Previous studies characterized BNR as occurring on average 43 ± 38 months following transplantation while the data collected in our study indicates that the time to the first incidence of BNR is 7.8 ± 7.5 months. The mechanism associated with BNR requires further understanding of its characteristics. Unfortunately not too many reports in the field looked at the treatment protocols for BNR. In our single center experience, who patients presented with heart failure symptoms were given more aggressive treatment, that is, ATG, IV methylprednisolone, and IVIG while patients without heart failure symptoms were mostly given prednisone bolus and taper only. As BNR is an infrequent phenomenon, of which there is considerable debate regarding its validity, further work needs to be instigated on whether there is a correlation between different factors and BNR episodes. Overall, from this set of data, it is inconclusive as to whether or not there are any characteristics of BNR that differs from other types of rejections.

A point of interest in this study is the patient who experienced two episodes of BNR. Due to the small sample size, it is uncertain whether having one episode of BNR increases the risk of recurrent BNR episodes. During the first BNR episode, this patient experienced a return to normalized LVEF within 90 days of onset. In contrast, it took the patient 6 months to experience a return to normalized LVEF (51%) during the second BNR episode. For the first episode of BNR, there was a favorable return to normal cardiac function following treatment with oral corticosteroids bolus and taper. In the case of the second episode of BNR, IVIG was given instead which led to an increase in LVEF, although it took a longer time (6 months) for the patient to experience a return to the range of normalized LVEF. Prior to experiencing normalized LVEF the patient's LVEF remained in the 40% range, fluctuating between 43% and 48%.

The repeat BNR patient was the only African Americans in this study and did not experience normalized LVEF at 90-day after second onset. African Americans have been reported to have a higher risk of rejection after transplant compared to any other race due to their unique metabolism in regard to immunosuppression [14, 15]. Thus their immunosuppression needs to be tailored differently. A possible immunosuppression regimen with respect to BNR could require more aggressive treatment, employing the use of ATG and IVIG in addition to steroids. Potential cause for this second onset of BNR could be the combined factor of this patient not receiving an adequate treatment for the second BNR and the ethnicity of the patient.

As cases of BNR tend to respond favorably to appropriate rejection therapy 2 (17%) of 12 BNR cases required inotropes and 7 (58%) BNR cases without heart failure symptoms were treated with oral steroid bolus and taper. Seven of 12 (58%) treated BNR cases resulted in a return to a normal LVEF of 57% ± 6% within a mean of 14 ± 10 days from the initial negative biopsy. Due to the small sample size, it is uncertain whether BNR should be considered as a new category in the biopsy grading scale or if it is merely difficult to diagnose based on endomyocardial biopsies alone. If it is the former, then mechanism needs to be elucidated. If it is the latter, then perhaps other methods for detecting rejection need to be considered.

## 5. Conclusion

BNR is a rare phenomenon (12 cases of BNR in a 10-year period) and can be a severe form of rejection in that there is cardiac dysfunction. However, this type of rejection is not apparent on biopsies due to either lack of knowledge regarding further types of rejection or other factors. Characteristics of these cases of rejection are described above with most cases responding favorablly to rejection therapy. A detailed mechanism of this type of rejection needs to be elucidated.

## Figures and Tables

**Table 1:** Baseline characteristics of the patients with treated BNR.

Baseline characteristics	BNR patients (*N* = 11)
Mean recipient age, years ± SD	58.1 ± 8.9
Mean donor age, years ± SD	31.2 ± 11.9
Recipient BMI, mean ± SD	26.7 ± 5.2
Mean ischemic time, mins± SD	191.3 ± 43.6
Primary reason for transplant, coronary artery disease as underlying diagnosis (%)	6/11 (55%)
Recipient race, African American (%)	1/11 (9%)
Female (%)	1/11 (9%)
Status 1 at listing (%)	10/11 (91%)
CMV mismatch (%)	1/11 (9%)
Diabetes mellitus (%)	4/11 (36%)
Treated hypertension (%)	7/11 (64%)
Previous blood transfusion (%)	0/11 (0%)
Insertion of ventricular assist device (%)	5/11 (45%)
Pretransplant PRA ≥ 10%	0/11 (0%)

**Table 2:** Immunosuppression at BNR onset.

Immunosuppression at BNR	BNR cases (*N* = 12)
Cyclosporine (%)	5/12 (42%)
Dose (mg ± SD)	194 ± 13
Trough level (ng/mL ± SD)	227 ± 66
Reason for switching from tacrolimus	Tacrolimus reduces seizure threshold
Tacrolimus (%)	6/12 (50%)
Dose (mg ± SD)	5.3 ± 1.8
Trough level (ng/mL ± SD)	10.0 ± 4.6
Sirolimus (%)	1/12 (8%)
Reason for switching from tacrolimus	Renal insufficiency
Azathioprine (%)	1/12 (8%)
Mycophenolate mofetil (%)	11/12 (92%)
Dose (mg ± SD)	1889 ± 821
Trough level (*μ*g/ML)	2.0 ± 1.6

**Table 3:** Characteristics of biopsy-negative rejection.

Characteristics	BNR cases (*N* = 12)
De novo antibody development posttransplant before BNR onset, %	3/12 (25%)
Donor-specific antibody, %	1/3 (33%)
LVEF % ± SD	
At discharge of initial hospitalization for heart transplant	61% ± 6%
Last LVEF before BNR onset	53% ± 6%
At BNR presentation	34% ± 10%
Cases with 1st year BNR, %	11/12 (92%)
No treated rejection before BNR, %	11/12 (92%)*
Biopsy features before BNR (*n* = 106)	
Biopsy cellular rejection episodes (1A, 1B, or 1R), %	25/106 (23%)
Biopsy antibody-mediated rejection episodes (1), %	1/106 (0.9%)
Treatment	
Require inotropes, %	4/12 (16.7%)
IV methylprednisolone followed by prednisone bolus and taper, %	4/12 (33%)
ATG, %	3/12 (25%)
IVIG, %	3/12 (25%)
Oral steroid treatment non-CHF cases, %	7/12 (58%)
Outcome	
Cases with normalized LVEF, %	7/12 (58%)
Mean LVEF at recovery, LVEF % ± SD	57% ± 6%
Mean days to recovery, days ± SD	14 ± 10
Negative (ACR 0R, AMR 0) biopsy result after treatment, %	10/12 (83%)

*The only case that represented the patient who had two BNRs during followup.
